# The Refraction of the Eye and the Anomalies of the Ocular Muscles

**Published:** 1904-03

**Authors:** 


**The Refraction of the Eye and the Anomalies of the Ocular Muscles.** By KENNETH CAMPBELL, M.B. Pp. 214. London: Baillière, Tindall and Cox. 1903.

The author in his preface says: "True science is itself simple, and should be explained in as simple and definite language as possible," and with this text before him he has brought out a work that for simplicity in explanation seems to be everything that can be required. He has selected that portion of ophthalmic surgery that is usually a difficulty to the ordinary student of medicine. With this work before him no one should be at a loss to grasp the whole theory of refraction.

